# Anabolic Androgenic Steroid Use Patterns and Steroid Use Disorders in a Sample of Male Gym Visitors

**DOI:** 10.1159/000528256

**Published:** 2023-02-02

**Authors:** Tjeerd Idger de Zeeuw, Tibor Markus Brunt, Jan van Amsterdam, Katinka van de Ven, Wim van den Brink

**Affiliations:** ^a^Mainline, Amsterdam, The Netherlands; ^b^Department of Psychiatry, Amsterdam University Medical Center, University of Amsterdam, Amsterdam, The Netherlands; ^c^Criminology, Faculty of Humanities, Arts, Social Sciences, and Education (HASSE), UNE, Armidale, New South Wales, Australia; ^d^Human Enhancement Drugs Network (HEDN), Armidale, New South Wales, Australia

**Keywords:** Anabolic androgenic steroids, Dependence, DSM-5, Substance use

## Abstract

**Introduction:**

The use of anabolic androgenic steroids (AAS) and other image- and performance-enhancing drugs is a growing public health concern. AAS use is associated with various physical and mental harms, including cardiovascular risks, cognitive deficiencies, and dependence. The aim of this study was to determine whether patterns of AAS use and other variables are associated with the presence of an AAS use disorder (AASUD).

**Methods:**

An online survey was completed by 103 male AAS consumers visiting gyms. The association of different patterns of AAS consumption (cycling vs. continuous forms of AAS use), psychoactive substance use, mental health disorders, and sociodemographic variables with moderate-severe AASUD (fifth edition of the Diagnostic and Statistical Manual of Mental Disorders ≥4 criteria) was investigated. The associations between duration of AAS use and the AAS dose with moderate-severe AASUD were investigated using logistic regression analysis with moderate-severe AASUD as the dependent variable.

**Results:**

Moderate-severe AASUD was present in 25 (24.3%) of the participants. AAS consumers meeting criteria for moderate-severe AASUD, compared to those that did not, in the last 12 months reported a longer duration of AAS use (in weeks), a higher average AAS dose (mg/week), and a greater number of AAS side effects. Duration of AAS use and the AAS dose were the only independent predictors, with an increase of 3.4% in the probability of moderate-severe AASUD with every week increase of the duration of AAS use in the last year (*p* < 0.05) and an increase in moderate-severe AASUD of 0.1% with every 10 mg increase in the average AAS dose per week (*p* < 0.05), respectively.

**Conclusion:**

Our findings show that moderate-severe AASUD is relatively frequent among male AAS consumers and is positively associated with the duration and average dose of AAS use in the last 12 months.

## Introduction

Anabolic androgenic steroids (AAS) can be medically prescribed for the treatment of delayed puberty and other medical problems caused by testosterone deficiency. Traditionally, nonmedically prescribed AAS were used by competitive weightlifters, powerlifters, and bodybuilders to gain muscle mass and strength to increase their performance. However, since the 1970s, AAS are increasingly used by recreational athletes to enhance physical appearance. Since then, in addition to AAS, a wide range of other substances, together termed “image- and performance-enhancing drugs” (IPEDs), including growth hormones and fat loss drugs, are used to alter physical performance or appearance [1, 2].

There is increasing evidence that AAS use is a growing public health concern [3], with an estimated global lifetime prevalence in the general population of 3.3% (95% CI: 2.8–3.8): in males significantly higher than in females (6.4% vs. 1.6%) [4]. The prevalence of AAS use is relatively high in Europe, North America, the Middle East, Oceania (Australia and New Zealand), and South America (Brazil) and relatively low in Africa and Asia [5]. The prevalence of AAS use is probably underestimated because these estimates are generally based on self-report data and because of the illegal nature of the supply and the secretive nature of their use [6]. In 2012/2013, about two-thirds of new clients of Needle and Syringe Programmes (NSP) in the UK were AAS consumers [7]. In 2009, a Dutch study concluded that 8.2% of 718 members of fitness centers used IPEDs, mainly AAS and stimulants [8], whereas more recently, another study among 2269 male gym visitors reported that 9.0% used AAS [9].

AAS consumers with a high cumulative history of AAS exposure are at risk for various physical problems [10], including hypogonadism [11], cardiovascular conditions [12, 13], and cognitive deficiencies [14]. In addition, AAS use is associated with the use of (other) illicit substances and AAS dependence disorder [15]. Although this disorder has been proposed and recognized by some [15, 16], it is important to note that psychiatric classification systems do not recognize AAS dependence as a mental disorder. For this reason, Kanayama, et al. [17] decided to use a slightly adapted version of the existing criteria for dependence according the fourth edition of the Diagnostic and Statistical Manual of Mental Disorders (DSM-4) with the restriction that AAS dependence is only present if there is a maladaptive pattern of AAS use associated with clinically significant impairment or distress, manifested by three or more of the seven DSM-4 criteria. According to Kanayama et al. [17], AAS dependence may arise when AAS use is continued despite surging prominent adverse medical and psychiatric effects.

Based on seven different studies, Kanayama et al. [17] concluded that about 30% of illicit AAS consumers developed AAS dependence. In an online survey among regular US visitors of Internet discussion boards about fitness, bodybuilding, weightlifting, and steroid use, 23.4% of 479 AAS consumers met criteria for AAS dependence [18]. A follow-up study confirmed these findings and showed higher AAS doses, higher quantity of agents, shorter periods without AAS consumption, and longer lifetime duration of AAS use in AAS-dependent consumers compared to AAS-nondependent consumers [19].

AAS dependence might be related to specific patterns of AAS use or doses used. To avoid negative health effects associated with continuous AAS consumption, some AAS consumers apply “cycles” with periods of AAS use interrupted by regular breaks with no AAS use that generally last at least as long as the periods AAS were used [20]. Other AAS consumers apply some form of continued use which, by varying degrees, may be sustained by positive effects during use, like the desired increase in muscle mass and feelings of confidence and well-being and, on the other hand, the avoidance of negative side effects, such as hypogonadal symptoms [21] that occur after AAS use and that may withhold many AAS consumers from cessation [22].

In the most recent version of the Diagnostic and Statistical Manual of Mental Disorders (DSM-5) [23], substance abuse and dependence are merged into one substance use disorder (SUD) with different levels of severity according to the presence of the number of criteria, ranging from a mild (2–3 of 11 criteria) through a moderate (4–5 of 11 criteria) to a severe (≥6 of 11 criteria) SUD. In this paper, we use the DSM-5 diagnostic criteria for SUD, specifically adjusted for AAS use, to assess the presence of a moderate or severe “AAS use disorder” (AASUD), i.e., participants meeting ≥4 (adjusted) DSM-5 SUD criteria.

Currently, there are no data in the literature about the relationship between AAS use patterns, duration of use, and the presence of AAS dependence or AASUD. Regarding the AAS dose, several studies have noted that dependent consumers took significantly more AAS than nondependent consumers in terms of total dose [19], total number or length of AAS cycles, and cumulative duration of AAS use [24]. We executed an online survey among AAS-using male gym visitors to investigate the prevalence of moderate-severe AASUD (≥4 DSM-5 criteria) and to detect variables associated with AASUD, including the pattern of AAS use, doses of AAS that were used (in mg/week in the last 12 months), duration of AAS use (weeks in the last 12 months), AAS side effects, psychoactive substance use, and the presence of mental disorders [19, 25]. Sociodemographic variables and the use of supplements were also investigated.

## Methods

### Study Population

This study is using a convenience sample of IPED consumers recruited in the Netherlands. Between December 12, 2019, and April 1, 2020, participants were contacted through social media (Facebook, Twitter), the harm reduction agency “Mainline” (www.mainline.nl), and the most visited Dutch forum for strength sport and bodybuilding (www.bodybuilding.nl). In addition, participants were recruited by posters and flyers in fitness centers and during the biggest strength sports and bodybuilding event in Belgium and the Netherlands (S.A.P. Cup: www.muscletotaal.nl/sap-cup). Participation in the survey was open for men and women aged 18 years or older. In the present study, participants were included if they were 18 years or older, had used AAS in the past 12 months, and had answered the 11 questions to assess the presence of an SUD (which equals completion of at least 70% of the survey questions).

The survey was carried out in accordance with the Code of Ethics of the World Medical Association (Declaration of Helsinki). Participants provided informed consent before participation in the survey. Responses were not traceable to a specific person, and no personal data were collected. Optionally, participants could leave an e-mail address after the survey to participate in the raffle of ten books. The collected data were stored in a secure folder encrypted with AES-256 encryption and maintained by Mainline with the encryption key solely in the possession of the principal investigators.

### Measurements

The online survey was designed in SurveyMonkey® (www.surveymonkey.com) and in total consisted of 76 items. Survey items related to either the participant's behavior and experiences in the past 12 months or the present situation, unless explicitly stated differently, measured the following variables: demographics (age, gender, educational level, working and partner status), sports practice (main sport practiced, intensity and frequency of training, competitive involvement, main sporting goal), supplement use, AAS and other IPED use (types of AAS used, frequency of use, route of administration, dose, pattern of AAS use, types of IPEDs used), side effects experienced, and psychoactive substance use. In addition, for mental health, participants were asked about their body image satisfaction and whether they ever or currently had one or more of the following conditions: ADHD, anxiety disorder, depression, eating disorder, psychosis, and/or substance dependence. Age of first occurrence of the mental condition and age of first AAS use were additionally asked. Participants who had a cyclic pattern of AAS use were asked to rate their mental well-being “on” and “off cycle” on a scale ranging from 0 to 10. A copy of the survey is included in online supplement S1 (for all online suppl. material, see www.karger.com/doi/10.1159/000528256).

AASUD was assessed by 11 yes/no questions regarding the presence of the DSM-5 diagnostic criteria for SUD but adjusted for AAS use [17]. In addition, the new “craving” criterion of DSM-5 (item 4) was split to make the item applicable in the context of both intermittent and continuous patterns of AAS use. In the current study, only participants with a moderate-severe DSM-5 AASUD are regarded to have a clinically relevant disorder, i.e., participants meeting ≥4 DSM-5 SUD criteria with AAS as the substance of abuse. Participants with a positive response on only 2 or 3 DSM-5 SUD criteria are not regarded to have a clinically relevant AASUD because this may result in a high number of false positives, i.e., the inclusion of AAS consumers reporting some problems with their AAS use without meeting clinical significant levels of a mental disorder.

### Statistical Analysis

Cases with a missing value on a variable were omitted from analyses that included the variable. Adjusted sample sizes for these cases were reported in the tables displaying the results. For the description of the study population, the normally distributed variables were expressed as means with standard deviations and for skewed distributed variables, the medians were calculated with interquartile ranges. The relationships between AASUD (yes vs. no) with demographic factors, sports characteristics, AAS use patterns, duration and quantity of use (AAS and other IPEDs), mental and physical health, and lifestyle factors were explored using, respectively, the Fisher-Freeman-Halton Exact test, Fisher's Exact test, and the independent samples *t* test or Mann-Whitney U test for significance. A correlation analysis was done to explore the relations of the presence or absence of moderate-severe AASUD (DSM-5 ≥4) and the number of AASUD criteria with age, substance use, (non-AAS) substance dependence, mental health disorders, IPED use other than AAS, and past physical or sexual abuse, based on the literature [19, 25, 26, 27]. Variables that were significantly associated with moderate-severe AASUD were investigated further in a logistic regression analysis with moderate-severe AASUD as the dependent variable. Significance was determined at *p* value <0.05. Statistics was performed with SPSS software, version 28.

## Results

### Sample

Of the 189 participants, 133 (70.4%) reported ever use of AAS. Three women, 16 men who had not used AAS in the past 12 months, and 11 men who completed less than 70% of the questions were excluded. The final sample consisted of 103 men.

### Characteristics of the Participants

The first column of Table 1 summarizes the demographic and physical training characteristics, AAS use patterns, psychoactive substance use, and mental health status of the 103 AAS consumers. Participants were all male with a mean age of 31 years, were generally well educated (intermediate/higher education; 78.6%, *n* = 81), and bodybuilding was the sport most frequently practiced (69.9%, *n* = 72).

### Patterns of AAS Use

Participants were classified according to three patterns of AAS use over the past 12 months: a continuous stable dose of AAS (*n* = 9, 8.7%), a continuous use pattern where “blasts” and “cruises” of high and lower AAS doses were alternated (*n* = 44, 42.7%), and a third pattern in which periods of AAS use and no AAS use were “cycled” (*n* = 50, 48.5%). Online supplementary Table S2 shows the specific doses and durations of AAS use for each of the three patterns.

### Prevalence of No, Mild, Moderate, and Severe AASUD

On average, AAS consumers reported the presence of 2.2 of the 11 DSM-5 AASUD criteria. No AASUD (<2 DSM-5 criteria) and mild AASUD (2–3 DSM-5 criteria) were observed in 37 (35.9%) and 41 (39.8%) participants, respectively. Moderate or severe AASUD was observed in 25 participants (24.3%): 13 (12.6%) with moderate AASUD (4–5 DSM-5 criteria) and 12 (11.7%) with severe AASUD (≥6 DSM-5 criteria). Table 2 depicts the prevalence of all DSM-5 criteria adapted for AAS use for the subgroups without (*n* = 78) and with (*n* = 25) moderate-severe AASUD and for the total sample (*N* = 103).

### Differences between Participants without and with AASUD

The second and third columns of Table 1 show the characteristics of the subgroups without (*N* = 78) and with (*N* = 25) moderate-severe AASUD. Compared to those without moderate-severe AASUD, participants with moderate-severe AASUD spent less time (in minutes a week) on training (*p* = 0.035) and less often competed as an athlete (currently or in the past) (*p* = 0.015). AAS consumers with and without moderate-severe AASUD in the last 12 months also significantly differed in their duration of AAS use in weeks (*p* = 0.019) and in their AAS dose in mg/week (*p* = 0.034). In the last 12 months, both oral and injectable AAS were taken for more weeks by AAS consumers with moderate-severe AASUD versus AAS consumers without moderate-severe AASUD (*p* = 0.003 and *p* = 0.039). Furthermore, AAS consumers with moderate-severe AASUD more frequently (*p* = 0.038) used (non-prescribed) insulin and/or DNP, two IPEDs with a high-risk profile [28, 29]. AAS consumers with moderate-severe AASUD versus AAS consumers without moderate-severe AASUD in the last 12 months also reported to have experienced significantly more side effects (*p* = 0.017). The pattern of AAS use was not associated with moderate-severe AASUD (*p* = 0.120). The higher prevalence of participants with a “blast-and-cruise” pattern of AAS use compared to those with a “cycling” AAS use pattern (60.0%, *n* = 15 vs. 32.0%, *n* = 8) among those with moderate-severe AASUD was not significant (*p* = 0.055 in the post hoc test).

Psychoactive substance use in the last 12 months and a current or past mental disorder were reported by, respectively, 44 (44.0%) and 40 (40.8%) participants. About three-quarters of participants (76.0%) were fairly or very satisfied with their physical appearance. No differences were reported by AAS consumers with and without moderate-severe AASUD in the use of psychoactive substances or alcohol and in mental health parameters. In online supplementary Tables S3 and S4, additional information can be found on participants' use of substances and mental health status.

### Logistic Regression Analyses to Identify the Correlates of AASUD

Variables were tested to examine their association with moderate-severe AASUD. Online supplementary Table S5 shows the correlation matrix of these variables. AAS dose in the last 12 months, duration of AAS use, AAS side effects, and lifetime mental disorder were significantly correlated with moderate-severe AASUD and/or the total number of AASUD criteria. Logistic regression analyses were then performed to identify the variables that were independently associated with the diagnosis moderate-severe AASUD. The variables linked to AAS use (AAS dose in the last 12 months in mg/week and duration of AAS use in weeks) showed considerable overlap with each other and with AAS side effects. To prevent collinearity problems, we excluded AAS side effects from the logistic models and tested separate logistic regression models for the relationship between the average AAS dose in the last 12 months and AAS use duration with moderate-severe AASUD. Calculated power for these analyses at this sample size was 0.826. Age and the lifetime presence of a mental disorder were included in the logistic models. Results are shown in Table 3. In the first logistic model (Table 3), with a total explained variance of 10.0%, duration of AAS use over last 12 months, but not age or mental health disorder, independently predicted moderate-severe AASUD with an increase of 3.4% in the probability of moderate-severe AASUD with an increase of every week of AAS use (*p* < 0.05). The second logistic regression model (Table 4) with a total explained variance of 13.8% showed that the AAS dose, but not age or mental health disorder, independently predicted moderate-severe AASUD with an increase of 0.1% in the probability of moderate-severe AASUD with the increase of 10 mg of AAS per week (*p* < 0.05).

### Additional Analyses to Test the Stability of the Associations

Additional explorative analyses showed that the average dose used during AAS consumption was not associated with moderate-severe AASUD (*p* > 0.05; online suppl. Table S6A). In a model with the average dose used during AAS consumption, age, and lifetime mental disorder, the duration of AAS use remained positively associated with moderate-severe AASUD (odds ratio = 1.032, *p* < 0.05). The explained variance of this model was 14.9% (online suppl. Table S6B). The strength or significance of the association (odds ratio = 1.032, *p* < 0.05) was not affected when continuous AAS consumers on non-supraphysiological doses (<200 mg/week. last 12 months, *n* = 4) were excluded from the model. To test whether exclusion of 11 participants with less than 70% of the questions answered may have influenced the results, this group was compared with included participants (*N* = 103) on demographic variables, supplement use, age of first use of AAS, and duration of AAS use in the last 12 months (missing values precluded further comparisons), and no group differences were found (results not shown).

## Discussion

The main findings of the current study are that 24.3% of our study sample of male gym goers meet (self-reported) criteria for a moderate-severe AASUD (≥4 DSM-5 criteria) and that the duration of AAS use in the last 12 months and the AAS dose in mg/week in the last 12 months are independently associated with moderate-severe AASUD. Every week increase of the duration of AAS use is associated with an increase of 3.4% in the probability of moderate-severe AASUD. Every 10 mg increase of the AAS dose per week is associated with an increase of 0.1% in the probability of moderate-severe AASUD. The association between duration of AAS use and moderate-severe AASUD was independent from the average (supraphysiological) dose used during AAS consumption.

The prevalence of moderate-severe AASUD of 24% is in line with earlier results of about 30% of all AAS consumers developing AAS dependence, based on the DSM-3-R/DSM-4 criteria for dependence (≥3 of 7 criteria) [16] and is in contrast with some previous reviews, stating that dependence liability of AAS is probably low [30]. A recent review, including data from 10 studies (total *N* = 1,247 AAS consumers), found a mean prevalence of AAS dependence across all studies of 32.5% (95% CI: 25.4–39.7), with a median of 29.5% [15]. The relatively lower prevalence of moderate-severe AASUD in the current study is mainly caused by the low prevalence of moderate-severe AASUD in the group of “cycling” AAS consumers (16.0%, *n* = 8), whereas moderate-severe AASUD is numerically overrepresented (34.1%, *n* = 15) in the group of continuous “blast-and-cruise” AAS consumers, with 32.0% versus 60.0%, respectively, within the group of AASUD. This calls for more attention to the potential role of chronic “blast-and-cruise” patterns of AAS use in the etiology of AASUD. This is especially important, given the recent increase in the “blast-and-cruise” pattern of AAS use. In 2007, in an online survey among 1,955 male AAS consumers, 5.0% indicated to have taken AAS continuously for the entire year [26], whereas little than a decade later, a similar study found that nearly half (47.32%) of the 2,385 AAS consumers reported a continuous “blast-and-cruise” use pattern [22]. This is important as with long-term continuous AAS use, it may be increasingly difficult to stop the use of AAS since the consumer will be confronted with the consequences of a prolonged disruption of endogenous testosterone production [11, 21].

To the best of our knowledge, this is the first study in which (slightly adjusted) DSM-5 criteria were used to assess the presence of AASUD. It should be noted however that AAS differ from traditional addictive substances, like cocaine and heroin, in that they produce little immediate reward or acute intoxication. The high rate of positive response to the criterion “spending a lot of time planning anabolic steroid use and/or obtaining anabolic steroids” may indicate the presence of a deliberate and regulated use pattern, instead of a loss of control over use. This pattern, unlike psychoactive substance use that is rather influenced by impulses, is regarded a typical feature of AAS use [26] and in itself not necessarily maladaptive or cause of distress. This finding is also in line with a recent study showing that time spent on activities related to the use of AAS was the symptom with the smallest effect on a DSM-4 symptom network of AAS dependence, whereas continuing AAS use despite physical and/or mental problems was the most central symptom [25]. Psychometric assessments like these are, therefore, essential to evaluate the criteria for AASUD, including the identification of the most central criteria that relate to the most typical and relevant symptoms of AASUD [25, 31].

Despite these definition issues, animal studies suggest that AAS can induce AAS dependence [32] and that AAS is a critical modulator of executive functions [33] and impairs behavioral flexibility and increases compulsivity [34]; findings that were corroborated in recent human studies [25, 35]. However, unlike many addictive substances, AAS do not acutely stimulate dopamine release in the nucleus accumbens [36]. One of the alternative explanations given for chronic and compulsive AAS consumption in conjunction with high intensity bodybuilding is social physical anxiety and negative perception of physical appearance [37]. However, in the current study, anxiety and dissatisfaction with one's physical appearance were infrequent in both AAS consumers with and without moderate-severe AASUD and thus unlikely to be an explanation for the compulsive consumption of AAS.

The current study has both strengths and limitations. The main strengths of the study are the detailed measurement of the different patterns of AAS use and the use of a broad range of variables as potential confounders for the development of moderate-severe AASUD. The main limitations are the unstructured sampling frame and the exclusive use of self-reported information and thus the risk of overreporting of AASUD criteria and mental disorders. However, the prevalence data in the current study are generally consistent with similar data from other studies using different recruitment and assessment procedures.

Furthermore, it is worth noting that the exclusion from the study of 16 AAS consumers who had not used AAS in the past 12 months may have influenced differences on some level between AAS consumers with and without moderate-severe AASUD. Exclusion of records, instead of imputation of missing values, for those participants with less than 70% of the questions answered (*n* = 11) may have also affected these differences due to a decrease of statistical power.

In the present study, AAS doses were estimated by adding up the self-reported doses in milligrams of different types of oral and injectable AAS. It should be stressed that this procedure does not acknowledge differences between AAS types in negative and positive effects and does not consider that the declared and the actual content or concentration of AAS from the black market often differ considerably [11, 27].

A potential reason for concern is the somewhat “circular” nature of the associations of moderate-severe AASUD with duration of AAS use and the AAS dose as the definition for moderate-severe AASUD includes criteria that relate to AAS use duration and dose. However, the key element in these criteria is loss of control, craving, and AAS use to avert withdrawal symptoms, and neither duration of use nor dose are in itself criteria that determine AASUD. As was pointed out earlier, the cross-sectional design of the present study prevents inferences about causality, and future studies with a prospective study design are needed to explore the hypotheses brought forward in this paper.

## Conclusion

Taken together, we found that the duration of AAS use (in weeks in the past 12 months) and AAS dose (in mg/week in the past 12 months) are associated with the presence of moderate-severe AASUD and that these effects remained significant after controlling for age and lifetime mental disorders. We conclude that AASUD could be a frequent complication in chronic, high-dose AAS consumers. Prospective research is needed to follow mental and physical changes over time in chronic AAS consumers and to identify those AAS consumers who make the transition from intermittent to chronic AAS use and, possibly, (moderate-severe) AASUD. Additionally, more research is required to aid chronic AAS consumers as the effectiveness of treatments for (moderate-severe) AASUD at present is undetermined and there is only scarce evidence for the benefits of interventions to reduce or stop the consumption of AAS [38]. Furthermore, to aid those with increased health risks due to chronic AAS use, healthcare should accommodate the specific needs of chronic AAS consumers [39]. However, at present, expertise and clinical guidelines in this domain are largely absent [40].

## Statement of Ethics

This study was executed according to the Code of Ethics of the World Medical Association (Declaration of Helsinki). According to Dutch law, ethical approval of the study was not required since (a) the survey was filled out fully anonymously, (b) participants were aged 18 years or above, (c) participants did not belong to a vulnerable group, (d) the questions posed to the participants were not burdensome with regard to completing the questions nor were the questions intimate about sexual behavior or psychological well-being, and (e) all participants provided written informed consent to use the information for research purposes before filling the questionnaire.

## Conflict of Interest Statement

The authors have no conflicts of interest to declare.

## Funding Sources

No funding was received for this study or the conception of this paper.

## Author Contributions

Tjeerd Idger de Zeeuw executed the study, assembled the database, analyzed the database, performed statistical analyses, and co-wrote the draft. Tibor Markus Brunt analyzed the database, performed statistical analyses, and wrote the draft. Jan van Amsterdam co-wrote the draft and reviewed the draft. Wim van den Brink checked and corrected statistics, critically reviewed, and corrected the final draft. Katinka van de Ven reviewed the final draft.

## Data Availability Statement

All data generated or analyzed during this study are included in this article and its online supplementary material. Further inquiries can be directed to the corresponding author.

## Supplementary Material

Supplementary data

Supplementary data

Supplementary data

Supplementary data

Supplementary data

Supplementary data

## Figures and Tables

**Table 1:** Characteristics of the total sample (*N* = 103) and the subgroups without (*N* = 78) and with (*N* = 25) AAS use disorder (AASUD; ≥4 DSM-5 criteria)

Variable^a^	Total sample (*N* = 103)	No AASUD (*N* = 78)	AASUD (*N* = 25)	*p* value
Age	31.2 (9.3)	31.37 (9.99)	30.76 (7.05)	0.777
Starting age of AAS use	25.2 (8.4)	25.91 (9.15)	22.84 (5.08)	0.113
Civil status, *n* (%)				
Partner, living together	44.0 (42.70)	34.0 (43.60)	10.0 (40.00)	0.835
Partner, not living together	23.0 (22.30)	18.0 (23.10)	5.0 (20.00)	
No partner or spouse	36.0 (35.00)	26.0 (33.30)	10.0 (40.00)	
Education, *n* (%)				
Elementary	7.0 (6.80)	4.0 (5.10)	3.0 (12.00)	0.071
Lower vocational or lower secondary education	15.0 (14.60)	12.0 (15.40)	3.0 (12.00)	
Intermediate vocational or intermediate/higher secondary education	37.0 (35.90)	24.0 (30.80)	13.0 (52.00)	
Higher vocational education or university	44.0 (42.70)	38.0 (48.70)	6.0 (24.00)	
Employment, *n* (%)				
Student or pupil	14.0 (13.60)	10.0 (12.80)	4.0 (16.00)	0.594
Unemployed or incapacitated	5.0 (4.90)	3.0 (3.80)	2.0 (8.00)	
Employed or runs his own business	84.0 (81.60)	65.0 (83.30)	19.0 (76.00)	
Main sports activity				
Bodybuilding, *n* (%)	72.0 (69.90)	55.0 (70.50)	17.0 (68.00)	0.890
Number of years active in main sports activity	9.0 (7.0–15.0)	9.0 (7.0–15.0)	10.0 (6.5–15.0)	0.865
Minutes of training a week	423.60 (176.6)	444.38 (186.64)	358.90 (122.85)	0.035*
Competitive athlete				
Yes (present or past), *n* (%)	31.0 (30.10)	28.0 (35.90)	3.0 (12.00)	0.015*
No	72.0 (69.90)	50.0 (64.10%)	22.0 (88.00%)	
Dietary supplements				
Number of supplement types used over 12 months	6.37 (3.86)	6.63 (3.67)	5.56 (4.38)	0.230
Pattern of AAS use, *n* (%)				
Continuous same dose	9.0 (8.74)	7.0 (8.97)	2.0 (8.00)	0.120
“Blast and cruise”	44.0 (42.72)	29.0 (37.18)	15.0 (60.00)	
“Cycling”	50.0 (48.54)	42.0 (53.85)	8.0 (32.00)	
AAS duration of use (weeks/12 months)	27.0 (16.0–52.0)	24.50 (15.00–52.00)	52.00 (22.00–52.00)	0.019*
AAS dose (mg/week) during AAS use (last 12 months)^b^	685.85 (500.00–950.00)	669.94 (500.00–950.00)	720.12 (589.50–991.00)	0.358
AAS dose (mg/week) over 12 months	385.77 (215.38–665.29)	367.31 (201.27–591.73)	638.86 (256.97–851.56)	0.034*
Oral AAS use (weeks/12 months)	4.0 (0.0–8.0)	3.5 (0.0–6.0)	8.0 (1.5–15.0)	0.003**
Injectable AAS use (weeks/12 months)	25.0 (16.0–52.0)	24.0 (12.0–52.0)	48.0 (20.0–52.0)	0.039*
Average no. of AAS in “stack” (i.e., used concomitantly)	2.0 (2.0–3.0)	2.0 (2.0–3.0)	3.0 (2.0–3.0)	0.583
IPEDs other than AAS (no. of types over 12 months)^c^^e^	3.57 (3.10)	3.35 (2.87)	4.25 (3.71)	0.219
Nonmedical insulin and/or DNP use in last 12 months^c^^e^, *n* (%)	14.0 (14.00)	7.0 (9.21)	7.0 (29.17)	0.038*
Concerns about the effect of AAS on long-term health^c^, *n* (%)	38.0 (38.00)	26.0 (34.20)	12.0 (50.00)	0.228
AAS side effects (no. over 12 months)^f^	4.51 (3.03)	4.10 (2.99)	5.76 (2.83)	0.017*
GP aware of AAS use^c^, *n* (%)	43.0 (43.00)	33.0 (43.40)	10.0 (41.70)	1.00
Blood test (laboratory analysis)^c^, *n* (%)	67.0 (67.00)	55.0 (72.40)	12.0 (50.00)	0.050
Smoking (daily)^c^, *n* (%)	16.0 (15.50)	11.0 (14.50)	5.0 (20.80)	0.651
Alcohol (any)^c^, *n* (%)	53.0 (53.00)	41.0 (53.90)	12.0 (50.00)	0.816
Drug use (any)^c^, *n* (%)	44.0 (44.00)	34.0 (44.70)	10.0 (41.70)	0.860
Satisfied with physical appearance^c^^g^, *n* (%)	76.0 (76.00)	61.0 (80.30)	15.0 (62.50)	0.100
Mental disorder (any) (lifetime)^h^, *n* (%)	40.0 (40.80)	28.0 (37.80)	12.0 (50.00)	0.343
Addiction (any) (lifetime)^h^, *n* (%)	15.0 (15.30)	9.0 (12.20)	6.0 (25.00)	0.189
Consulted a psychologist or psychiatrist^i^, *n* (%)	12.0 (12.40)	8.0 (10.80)	4.0 (17.40)	0.470

Numbers in the table are number (percentage), mean (standard deviation), or median (interquartile range); significance of group differences were tested with the Fisher-Freeman-Halton Exact test, Fisher's Exact test, unpaired *T* test, and Mann-Whitney U test. DSM-5, Diagnostic and Statistical Manual of Mental Disorders (5th ed.); DNP, 2,4-dinitrophenol; mg, milligram.

a Items refer to the last 12 months, unless stated otherwise.

b For AAS consumers with a “blast-and-cruise” use pattern, this is the average of the dose during a “cruise” and during a “blast,” taking into account their respective duration.

c *N* = 100 (no AASUD = 76, AASUD = 24). ^d^IPEDs, image- and performance-enhancing drugs.

e Without doctor's prescription.

f *N* = 102 (no AASUD = 77, AASUD = 25).

g “Fairly” and “very” satisfied combined.

h *N* = 98 (no AASUD = 74, AASUD = 24).

i *N* = 97 (no AASUD = 74, AASUD = 23); # continuous same dose versus “blast and cruise”; ¥ continuous same dose versus “cycling.”

* Significant at *p* < 0.05.

** Significant at *p* < 0.01.

**Table 2:** Reported symptoms of AAS use disorder (AASUD) in the total sample (*N* = 103) and the subgroups without (*N* = 78) and with (*N* = 25) AASUD (≥4 DSM-5 criteria)

	In the last 12 months, have you … answer	No AASUD (*N* = 78) “yes”		AASUD (*N* = 25) “yes”		Total sample (*N* = 103) “yes”	
		*N*	%	*N*	%	*N*	%
1	Used anabolic steroids more often, for longer periods, and/or in higher amounts than intended?	9	11.5	15	60.0**	24	23.3
2	Made unsuccessful efforts to cut down or stop using anabolic steroids?	2	2.6	6	24.0**	8	7.8
3	Spent a lot of time planning anabolic steroid use and/or obtaining anabolic steroids?	24	30.8	19	76.0**	43	41.7
4	a) Regularly had a strong desire to use anabolic steroids again after using anabolic steroids? or b) Regularly had a strong desire to increase the dose of anabolic steroids, during periods of lighter doses?	23	29.5	21	84.0**	44	42.7
5	Regularly been unable to fulfill your obligations at work, study, or at home due to the use of anabolic steroids?	1	1.3	2	8.0	3	2.9
6	Used anabolic steroids when you knew it would cause or worsen problems in your relationships with others?	14	17.9	16	64.0**	30	29.1
7	Had to give up or reduce important social, occupational, or recreational activities because of the use of anabolic steroids?	2	2.6	5	20.0**	7	6.8
8	Used anabolic steroids while it repeatedly got you in potentially physically dangerous situations such as fights, speeding, or unprotected sex?	3	3.8	4	16.0	7	6.8
9	Used anabolic steroids when you knew that a physical or mental problem could return or worsen?	12	15.4	19	76.0**	31	30.1
10	a) Needed increasing doses of anabolic steroids to achieve the desired effect? or b) Noticed that the same dose of anabolic steroids had less effect than before?	6	7.7	14	56.0**	20	19.4
11	a) Felt very unwell after stopping anabolic steroids, with at least two of the following symptoms: depressed mood, severe fatigue, difficulty sleeping, loss of appetite, and/or loss of libido? or b) Used anabolic steroids to eliminate or avoid unpleasant symptoms that occur after stopping anabolic steroids?	16	20.5	20	80.0**	36	35.0

DSM-5, Diagnostic and Statistical Manual of Mental Disorders (5th ed.). Significance of group differences were tested with the Fisher's Exact test,

** significant at *p* < 0.01. Addendum: •The seven items with numbers in bold represent the DSM-4 criteria for dependence. The remaining four items represent the new, additional, DSM-5 criteria. All items were formulated to accommodate the specific features of AAS use. •Item 3 was rephrased compared to previous questionnaires measuring AAS dependence (e.g., Kanayama et al., 2009) to match the characteristics of AAS use. •The fourth of the eleven diagnostic criteria in DSM-5 was unsuitable for use with continuous AAS users as it measures the desire to use again. Therefore, item 4b was added to the questionnaire. •For item 8, situations were chosen that may possibly result from AAS's mental effects.

**Table 3:** Logistic regression analyses predicting AAS use disorder (AASUD; DSM-5 criteria ≥4)

Variable	β		Wald's χ^2^	df	Eβ (OR)	95% CI
Age, years	−0.006	0.029	0.049	1	0.994	0.940–1.052
AAS duration (weeks) over 12 months	0.034	0.014	5.735	1	1.034*	1.006–1.064
Lifetime mental disorder	0.361	0.478	0.572	1	1.435	0.563–3.660
Constant	−2.320	1.079	4.623	1	0.098	

OR, odds ratio; SE, standard error. Duration (weeks) of AAS use in the last 12 months as the predicting variable.

* Significant at *p* < 0.05; Nagelkerke *R*^2^ for this model: 0.101.

**Table 4:** Average dose (mg/week) in the last 12 months as the predicting variable

Variable	β	SE β	Wald's χ^2^	df	Eβ (OR)	95% CI
Age, years	−0.006	0.029	0.049	1	0.994	0.939–1.052
AAS dose (mg/week) over 12 months	0.001	0.000	4.726	1	1.001*	1.001–1.002
Lifetime mental disorder	0.361	0.228	2.205	1	1.404	0.897–2.196
Constant	−2.320	0.966	3.438	1	0.167	

AAS, anabolic androgenic steroids; DSM-5, Diagnostic and Statistical Manual of Mental Disorders (5th ed.); OR, odds ratio; SE, standard error. *N* = 98 (no AASUD = 74, AASUD = 24) due to missing cases (*n* = 5) for “lifetime mental disorder.”

* Significant at *p* < 0.05; Nagelkerke *R*^2^ for this model: 0.138.
