# Supplementary material for: Anabolic Androgenic Steroid Use Patterns and Steroid Use Disorders in a Sample of Male Gym Visitors
Source: Eur Addict Res. 2023 Feb 2;29(2):99–108. doi: 10.1159/000528256 (PMC10273855; doi:10.1159/000528256)
Supplement: Supplementary file 1 — Supplementary data [file ear-0029-0099-s01.docx]

**Supplement S1.** Copy of the Questionnaire Used in the Online Survey.

1. I am...

·       A man

·       A woman

·       Other, namely...

2. I am ... years old.

3. I live in...

·       The Netherlands

·       Belgium

·       Other E.U. country than the Netherlands or Belgium

·       Outside the E.U.

4. My highest level of education completed is...

·       No education

·       Primary education

·       Practical education (“Praktijkonderwijs”)

·       Vmbo

·       Mavo

·       Havo

·       Vwo

·       Mbo

·       Hbo

·       University

·       Other, namely...

5. I am...

·       A student

·       Unemployed

·       Employed in a paid job, self-employed, or/and a business owner

6. I have...

·       A partner I live with

·       A partner I don't live with

·       No partner

7. I practice...

If you practice multiple sports, select the one you spent the most time and energy on in the past 12 months.

·       Bodybuilding

·       Fitness

·       Weightlifting

·       Powerlifting

·       Strongman

·       CrossFit

·       Calisthenics

·       Athletics and/or running

·       Team sports (football, basketball, etc.)

·       Tennis, badminton or table tennis

·       Martial arts (MMA, boxing, etc.)

·       Cycling

·       Swimming

·       Other, namely...

·       No sport

(Participants that answer "no sport" proceed to question 12)

8. I started practicing this sport when I was ... years old.

9. In the past 12 months, I practiced this sport on average ... days a week.

10. In the past 12 months, on the days I practiced this sport, it on average was for ... minutes.

11. Do you compete in this sport, or did you compete in this sport in the past?

a) Yes, I am active as a competitive athlete

b) Yes, I *have been* active as a competitive athlete

c) No, I am not (or have not been) active in this sport as a competitive athlete

12. In the past 12 months, my *main* sporting and/or cosmetic goal was to...

·       Improve stamina/endurance

·       Recover from an injury

·       Build muscle

·       Get stronger

·       Burn fat

·       Maintain my shape (physical, strength and/or endurance)

·       Do well at a bodybuilding/physique competition

·       Do well at a (strength) sports competition

·       Other, namely...

·       I did not have a sporting and/or cosmetic goal

13. In the past 12 months, did you use dietary supplements?

·       Yes

·       No

(Participants that answer "yes" proceed to question 14, those that answer "no" proceed to question 15)

14. In the past 12 months, what dietary supplements^*^ did you use (tick all that apply)?

^*^ Do *not* count products whose sale as a dietary supplement is prohibited in the Netherlands.

·       Amino acids/BCAAs/glutamine

·       Beta-alanine

·       Caffeine (tabs/caps)

·       Carnitine

·       Creatine

·       Fat burner

·       Glucosamine, chondroitin, and/or MSM

·       HMB

·       Homeopathic and/or Ayurvedic products

·       Herbal extracts

·       Melatonin

·       MCT oil

·       N.O. booster (citrulline, arginine, etc.)

·       Omega-3/fish oil

·       Omega-3-6-9

·       Phenibut

·       Pre-workout

·       Probiotics

·       Protein powder (whey, casein, plant-based, etc.)

·       Testosterone booster and/or tribulus terrestris

·       Vitamins and/or minerals

·       Vitargo/ waxy maize/ maltodextrin

·       Weight gainer

·       Other, namely....

15. Do you currently use anabolic steroids or did you use anabolic steroids in the past?

·       Yes

·       No, but I'm considering to start using them

·       No, and I will not use them

(Participant that answer "yes" proceed to question 16, those that answer "no" proceed to question 61)

16. I started using anabolic steroids when I was ... years old.

17. In the past 12 months, did you use anabolic steroids?

·       Yes

·       No

(Participants that answer "yes" proceed to question 18, those that answer "no" proceed to question 61)

18. In the past 12 months^*^, I used anabolic steroids for a total of .... (1 - 52) weeks.

^*^ 12 months = 52 weeks

19. In the past 12 months, I used *oral*anabolic steroids (tablets, capsules) for a total of .... (1 - 52) weeks.

(Participants that answer 0 proceed to question 21, those that answer ≥1 proceed to question 20)

20. In the past 12 months, I used the following *oral*anabolic steroids (tick all that apply):

·       Chlorodehydromethylandrostenediol (Halodrol)

·       Chlorodehydromethyltestosterone (Turinabol)

·       Fluoxymesterone (Halotestin)

·       Mesterolone (Proviron)

·       Metenolone (Primobolan tabs)

·       Methandrostenolone, methandienone (Dianabol)

·       Oxandrolone (Anavar)

·       Oxymetholone (Anapolon, Anadrol)

·       Stanozolol (Winstrol tabs)

·       Testosterone undecanoate (Andriol)

·       Other, namely...

21. In the past 12 months, I used *injectable* anabolic steroids (ampoules, multi-dose vials) for a total of .... (1 - 52) weeks.

(Participants that answer 0 proceed to question 24, those that answer ≥1 proceed to question 22)

22. In the past 12 months, I used the following *injectable* anabolic steroids (tick all that apply):

·       Boldenone undecylenate (Equipoise)

·       Drostanolone enanthate

·       Drostanolone propionate (Masteron)

·       Metenolone enanthate (Primobolan Depot)

·       Mix/blends (Rip-Fast, Mass-Mix, Primotest, etc.)

·       Nandrolone decanoate (Deca-Durabolin)

·       Nandrolone phenylpropionate (NPP)

·       Stanozolol (Winstrol Depot)

·       Testosterone cypionate

·       Testosterone enanthate

·       Testosterone mix (Sustanon, Omnadren)

·       Testosterone propionate

·       Trenbolone acetate

·       Trenbolone hexahydrobenzylcarbonate (Parabolan)

·       Trenbolone enanthate

·       Trestolone acetate (MENT)

·       Other, namely...

23. In the past 12 months, I have shared syringes or needles.

·       Yes

·       No

24. In the past 12 months, I obtained my anabolic steroids from... (tick all that apply):

·       A pharmacy outside the Netherlands or Belgium

·       A pharmacy in the Netherlands or Belgium

·       A dealer

·       A friend

·       A trainer/coach

·       The internet

· Contacts at the gym

·       Myself (self made anabolic steroids)

·       Other, namely...

·       I prefer not to say

25. In the past 12 months, before I started using an anabolic steroid, I was aware of the possible side effects of that anabolic steroid.

·       Yes

·       No

26. In the past 12 months, I had anabolic steroids tested in a laboratory, or tested one or more anabolic steroids myself with a testing kit.

·       Yes

·       No

27. In the past 12 months, I sometimes had serious doubts as to whether an anabolic steroid I was using contained the exact substance and/or dose stated on the label.

·       Yes

·       No

28. In the past 12 months, I used several anabolic steroids *at the same time* ("stacking").

·       Yes

·       No

(Participants that answer "yes" proceed to question 29, those that answer "no" proceed to question 30)

29. In the past 12 months, I combined on average ... (2 – 12) types of anabolic steroids during anabolic steroid use.

30. Which description best reflects the way you used anabolic steroids in the past 12 months?

a) I alternated periods of anabolic steroid use and periods that I did not use anabolic steroids (*"cycling"*).

b) I alternated periods with higher and lower doses of anabolic steroids, without stopping the use of anabolic steroid (*"blast & cruise"*).

c) I used anabolic steroids in a more or less equal dose for the entire 12 months.

(Participants that answer "a" proceed to question 36, those that answer "b" proceed to question 32, and participants that answer "c" proceed to question 31)

31. In the past 12 months, I used on average ... mg of anabolic steroids *per week*.

Please note!
1) Oral and injectable anabolic steroids are added up.
2) The number of milligrams (mg) is requested here, not the number of milliliters (ml) (for injectable anabolic steroids).

Depending on the specific type of product, the number of mg per ml may differ for injectable anabolic steroids, e.g. with testosterone enanthate the concentration is often 250mg per ml.; 3ml per week then is equal to 750 mg per week.

(Participants that answered question 31 proceed to question 43)

32. In the past 12 months^*^, the period(s) that I used a *higher* dose of anabolic steroids (the *"blast"*) lasted on average ... (1 – 52) weeks.

^*^12 months = 52 weeks

33. In the past 12 months, during a *"blast",* I took on average ... mg of anabolic steroids *per week*.

Please note!
1) Oral and injectable anabolic steroids are added up.
2) The number of milligrams (mg) is requested here, not the number of milliliters (ml) (for injectable anabolic steroids).

Depending on the specific type of product, the number of mg per ml may differ for injectable anabolic steroids, e.g. with testosterone enanthate the concentration is often 250mg per ml.; 3ml per week then is equal to 750 mg per week.

34. In the past 12 months, the period(s) that I used a *lower* dose of anabolic steroids (the *"cruise")* lasted on average... (1 – 52) weeks.

35. In the past 12 months, during a *"cruise",* I took on average ... mg of anabolic steroids *per week*.

Please note!
1) Oral and injectable anabolic steroids are added up.
2) The number of milligrams (mg) is requested here, not the number of milliliters (ml) (for injectable anabolic steroids).

(Participants that answered question 35 proceed to question 43)

36. In the past 12 months, I did .... (1 – 12) *“cycles”* ^*^ of anabolic steroids.

^*^ A *"cycle"* refers to a (continuous) period of anabolic steroid use.

37. In the past 12 months^*^, a *cycle* lasted on average ... (1 – 52) weeks.

^*^12 months = 52 weeks

38. In the past 12 months, the period(s) I did *not use* anabolic steroids lasted on average ... (1 – 52) weeks.

39. In the past 12 months, during a *cycle* I took on average ... mg of anabolic steroids *per week*.

Please note!
1) Oral and injectable anabolic steroids are added up.
2) The number of milligrams (mg) is requested here, not the number of milliliters (ml) (for injectable anabolic steroids).

Depending on the specific type of product, the number of mg per ml may differ for injectable anabolic steroids, e.g. with testosterone enanthate the concentration is often 250mg per ml.; 3ml per week then is equal to 750 mg per week.

40. In the past 12 months, at the end of a cycle, I followed a *post cycle therapy* (PCT) protocol, in which I used HCG, SERMs (e.g. Nolvadex, Clomid), and/or aromatase inhibitors (e.g. Arimidex, Aromasin) to stimulate the recovery of my testosterone production.

·       Yes

·       No

41. In the past 12 months, *during* anabolic steroids use*,* I mentally felt ... *(Scale 0 - 10; 0=Very bad, 5=Neutral, 10= Very good)*

42. In the past 12 months, *after* anabolic steroid use, I mentally felt ... *(Scale 0 - 10; 0=Very bad, 5=Neutral, 10= Very good)*

43. I anticipate to use anabolic steroids again in the next 12 months.

·       Yes

·       No

44 - 54. In the past 12 months, have you… (answer options: *yes/no*):

1. Used anabolic steroids more often, for longer periods, and/or in higher amounts than intended?
2. Made unsuccessful efforts to cut down or stop using anabolic steroids?
3. Spent a lot of time planning anabolic steroid use and/or obtaining anabolic steroids?
4. a) Regularly had a strong desire to use anabolic steroids again after using anabolic steroids?

*or:*

b) Regularly had a strong desire to increase the dosage of anabolic steroids, during periods of lighter dosages?

1. Regularly been unable to fulfill your obligations at work, study, or at home due to the use of anabolic steroids?
2. Used anabolic steroids when you knew it would cause or worsen problems in your relationships with others?
3. Had to give up or reduce important social, occupational, or recreational activities because of the use of anabolic steroids?
4. Used anabolic steroids while repeatedly ending up in potentially physically dangerous situations such as fights, speeding, or unprotected sex?
5. Used anabolic steroids when you knew that a physical or mental problem could return or worsen?
6. a) Needed increasing dosages of anabolic steroids to achieve the desired effect?

*or:*

b) Noticed that the same dosage of anabolic steroids had less effect than before?

1. a) Felt very unwell after stopping anabolic steroids, with at least two of the following symptoms: depressed mood, severe fatigue, difficulty sleeping, loss of appetite, and/or loss of libido?

*or:*

b) Used anabolic steroids to eliminate or avoid unpleasant symptoms that occur after stopping anabolic steroids?

55. In the past 12 months, I planned my anabolic steroid use based on advice and/or information from... (tick all that apply)

·       A physician

·       A dealer

·       An informative website

·       An internet forum

·       A popular reference work (e.g. William Llewellyn's "Anabolics")

·       A trainer/coach/nutritionist

·       A training partner and/or friend

·       Scientific publications

·       Other, namely...

·       Nobody/nothing, I know everything I need to know

56. In the past 12 months, what side effects did you experience during and/or after the use of anabolic steroids (tick all that apply)?

·       Abscess/bacterial infection at injection site

·       Acne/pustules

·       Decrease in testicle volume

·       Anxiety

·       Aggression or severe irritability

·       Clitoromegaly (enlarged clitoris)

·       Depression or severe gloom

·       Erectile disfunction

·       Emotional lability/strong mood swings

·       Gynecomastia (male breast formation)

·       Jaundice

·       Hair growth face/body

·       High blood pressure

·       Hyperhidrosis (excessive sweating)

·       Baldness/loss of head hair

·       Fever

·       Libido issues (a decrease or increase in the desire for sex that is experienced as unpleasant)

·       Mania or hypomania

·       Nausea

·       Infertility

·       Tendon rupture

·       Pain and/or swelling at injection site

·       Psychosis

·       Insomnia

·       Voice changes

·       Stretch marks

·       Increase in body fat

·       Blood test values outside reference range (cholesterol, hematocrit, liver, etc.)

·       Fluid retention/edema

·       Other, namely...

·       I have not experienced any side effects

(Participants that answered "I have not experienced any side effects" proceed to question 59)

57. I did the following when side effects occurred... (tick all that apply)

a) Nothing

b) Contacted a general practitioner and/or medical specialist

c) Took medication against the side effects

d) Switched to other anabolic steroids

e) Reduced the dosage of anabolic steroids

f) Stopped anabolic steroid use

g) Other, namely...

(All participants with answer that does *not* include "b" proceed to question 58, participants with answer that *does* include "b" proceed to question 59)

58. I *did not* contact a general practitioner or medical specialist when side effects occurred because... (tick all that apply)

·       I didn't find the side effects serious enough

·       I assumed that the side effects would go away on their own

·       I already got advice from a coach or friend

·       I knew what to do against the side effects

·       I assumed a doctor would demand me to stop using anabolic steroid, and I didn't want to do that

·       I have no faith in doctors when it comes to their knowledge about the use of anabolic steroids

·       I didn't want to have to defend myself or be lectured by a doctor

·       I did not want anabolic steroids use to be named in my medical record

·       I was afraid of the financial consequences (deductible health insurance, etc.)

·       Other, namely...

59. My general practitioner knows that I use anabolic steroids.

·       Yes

·       No

60. I am concerned about the impact of my anabolic steroid use on my long-term health.

·       Yes

·       No

 61. In the past 12 months, I had blood tests done.

·       Yes

·       No

62. In the past 12 months, I donated blood to the blood bank

·       Yes

·       No

63. In the past 12 months, I measured or had my blood pressure measured.

·       Yes

·       No

64. In the past 12 months, which of the following medicines and/or performance-enhancing drugs did you take*without a doctor's prescription*(tick all that apply)?

·       Aromatase inhibitors (Arimidex, Aromasin, Femara)

·       Benzodiazepines/tranquilizers (e.g. diazepam, oxazepam)

·       Blood pressure lowering agents (excluding diuretics)

·       Bromocriptine/cabergoline

·       Clenbuterol

·       Clomid (clomiphene)

·       DHEA

·       Diuretics

·       DNP

·       ECA (ephedrine, caffeine, aspirin)

·       Ephedrine

·       Erectile dysfunction medication (e.g. Viagra, Cialis)

·       Finasteride/dutasteride

·       Growth Hormones (HGH)

·       HCG (e.g. Pregnyl)

·       HMG (menotropin)

·       IGF-1

·       Insulin

·       Ionamin/Iomax (phentermine)

·       Melanotan

·       Metformin

·       Modafinil

·       Nolvadex (tamoxifen)

·       Opioids (e.g. fentanyl, oxycodone)

·       Priligy (dapoxetine)

·       Prohormones

·       Reductil (sibutramine)

·       Research peptides (e.g. GHRP, MGF, PGF, PT-141)

·       Ritalin/Concerta (methylphenidate)

·       Roaccutane (isotretinoin)

·       SARMs

·       Salbutemol

·       Thyroid hormones (T3, T4)

·       Site enhancement oil (e.g. Synthol)

·       Sleep medication (e.g. temazepam, midazolam)

·       Yohimbine

·       Other, namely...

·       Of the drugs mentioned, I did not take any without a doctor's prescription

65. In the past 12 months, did you smoke cigarettes, rolling tobacco, pipe, cigars, or e-smoker?

·       Yes, daily

·       Yes, occasionally

·       No

66. In the past 12 months, did you drink alcohol?

·       Yes

·       No

(Participants that answer "yes" proceed to question 67, those that answer "no" proceed to question 68)

67. In the past 12 months, on one single occasion how often did you drink four (for females) or five (for males) glasses of alcohol or more?

·       0 times

·       1 – 4 times

·       5 – 8 times

·       9 – 12 times

·       > 12 times

68. In the past 12 months, did you use illicit drugs^*^?

·       Yes

·       No

·       I’d rather not answer that

^*^ Do *not* count legal psychoactive substances such as caffeine, nitrous oxide, or products from the smart shop.

(Participants that answer "yes" proceed to question 69, those that answer "no" proceed to question 71)

69. In the past 12 months, what illicit drugs^*^ did you use (tick all that apply)?

·       Amphetamine/speed

·       Cannabis

·       Cocaine

·       Designer drugs (e.g. mephedrone, 2C-B, 4-FA/4-FMP)

·       GHB

·       Hallucinogens (e.g. LSD, magic mushrooms, ayahuasca)

·       Heroin

·       Ketamine

·       Methamphetamine

·       Ecstasy/MDMA

·       Other, namely....

^*^ Do *not* count legal psychoactive substances such as caffeine, nitrous oxide, or products from the smart shop.

70. In the past *4 weeks*, what illicit drugs^*^ did you use (tick all that apply)?

·       Amphetamine/speed

·       Cannabis

·       Cocaine

·       Designer drugs (e.g. mephedrone, 2C-B, 4-FA/4-FMP)

·       GHB

·       Hallucinogens (e.g. LSD, magic mushrooms, ayahuasca)

·       Heroin

·       Ketamine

·       Methamphetamine

·       Ecstasy/MDMA

·       Other, namely....

^*^ Do *not* count legal psychoactive substances such as caffeine, nitrous oxide, or products from the smart shop.

71. How satisfied or dissatisfied are you with the appearance of your body?

·       Very satisfied

·       Reasonably satisfied

·       A little dissatisfied

·       Very dissatisfied

72. What mental disorder or condition do you have, or did you have in the past (tick all that apply)?

·       ADHD

·       Anxiety disorder or severe anxiety

·       Bipolar disorder

·       Depression or severe depressive symptoms

·       Eating disorder

·       Psychosis

·       Addiction (drugs, alcohol, gambling, gaming, sex, sports)

·       I prefer not to say

·       None of these disorders/conditions

(Participants that answer "none" or “I prefer not to say” proceed to question 74, participants that select one of the other answers proceed to question 73)

73. When I was ... years old, the mental disorder/condition occurred for the first time.

If you have more than one mental disorder/condition, please indicate the age at which one of these disorders/conditions occurred for the first time.

74. In the past 12 months, I have visited a psychologist and/or psychiatrist^*^.

·       Yes

·       No

·       I prefer not to say

^*^ The reason you have visited a psychologist and/or psychiatrist is not of importance here.

75. Have you ever experienced physical or sexual abuse?

·       Yes

·       No

·       I prefer not to say

76. Have you ever been sentenced to prison?

·       Yes

·       No

·       I prefer not to say
