# Supplementary material for: Anabolic Androgenic Steroid Use Patterns and Steroid Use Disorders in a Sample of Male Gym Visitors
Source: Eur Addict Res. 2023 Feb 2;29(2):99–108. doi: 10.1159/000528256 (PMC10273855; doi:10.1159/000528256)
Supplement: Supplementary file 2 — Supplementary data [file ear-0029-0099-s02.docx]

**Table S2.** Patterns of anabolic-androgenic steroid (AAS) use in the last 12 months (N=103).

| Pattern | N | % | Median (IQR) |
| --- | --- | --- | --- |
|  |  |  |  |
| *Continuous stable use (non-stop; same dose)* | 9 | 8.7 |  |
| AAS mg per week during AAS use |  |  | 258 (125 – 1376) |
| AAS mg per week over the last 12 mo. |  |  | 258 (125 – 1104) |
| Cumulative AAS (mg) over the last 12 mo. |  |  | 13,416 (6,500 – 57,408) |
|  |  |  |  |
| *Continuous Blast & Cruise use ^¥^* | 44 | 42.7 |  |
| AAS mg per week during blast |  |  | 1,000 (900 – 1,438) |
| Duration of blast (wks.) |  |  | 12.0 (10.2 – 16.0) |
| AAS mg per week during cruise |  |  | 219 (150 – 252) |
| Duration of cruise (wks.) |  |  | 12.0 (8.0 – 15.0) |
| AAS mg per week over the last 12 mo. |  |  | 626 (381 – 774) |
| Cumulative AAS (mg) over the last 12 mo. |  |  | 32,251 (19,800 – 40,251) |
|  |  |  |  |
| *Cycling use* ^§^ | 50 | 48.5 |  |
| AAS mg per week during cycle |  |  | 775 (507 – 1,078) |
| Duration cycle (wks.) |  |  | 10.0 (8.7 – 13.0) |
| Duration off-cycle (wks.) |  |  | 22.0 (11.7 – 40.0) |
| Number of cycles over the last 12 mo. |  |  | 2.0 (1.0 – 2.0) |
| AAS mg per week over the last 12 mo. |  |  | 301 (143 – 415) |
| Cumulative AAS (mg) over the last 12 mo. |  |  | 15,664 (7,425 – 21,600) |

IQR = interquartile range; ^¥^ continued AAS use, where higher (the “blast”) and lower doses (the “cruise”) are periodically alternated; ^§^ intermittent AAS use, where AAS use (the “cycle”) and no AAS use (the “off-cycle”) are periodically alternated.
