# Supplementary material for: Anabolic Androgenic Steroid Use Patterns and Steroid Use Disorders in a Sample of Male Gym Visitors
Source: Eur Addict Res. 2023 Feb 2;29(2):99–108. doi: 10.1159/000528256 (PMC10273855; doi:10.1159/000528256)
Supplement: Supplementary file 3 — Supplementary data [file ear-0029-0099-s03.docx]

**Table S3.** Use of substances in male anabolic-androgenic steroid (AAS) consumers (N=103).

| Variable | N | % | Mean (SD) |
| --- | --- | --- | --- |
| Used dietary supplements in the last 12 mo. | 97 | 94.2 |  |
| *Most frequently used dietary supplements* |  |  |  |
| Protein powder | 84 | 81.6 |  |
| Vitamins/minerals | 70 | 68.0 |  |
| Creatine | 67 | 65.0 |  |
| No. of dietary supplement types used in the last 12 mo. |  |  | 6.37 (3.86) |
| Used drugs in the last 12 mo. (n=100) | 44 | 44.0 |  |
| *Most frequently used drugs* |  |  |  |
| Cannabis | 26 | 26.0 |  |
| XTC/MDMA | 24 | 24.0 |  |
| Amphetamine | 16 | 16.0 |  |
| GHB | 16 | 16.0 |  |
| No. of drug types used in the last 12 mo. (n=100) |  |  |  |
| 0 | 56 | 56.0 |  |
| 1 - 2 | 24 | 24.0 |  |
| 3 - 4 | 8 | 8.0 |  |
| ≥ 5 | 12 | 12.0 |  |
| Used drugs in the last 4 wks. (n=100) | 30 | 30.0 |  |
| *Most frequently used drugs* |  |  |  |
| Cannabis | 16 | 16.0 |  |
| GHB | 9 | 9.0 |  |
| Cocaine | 9 | 9.0 |  |
| No. of drug types used in the last 4 wks. (n=100) |  |  |  |
| 0 | 70 | 70.0 |  |
| 1 - 2 | 25 | 25.0 |  |
| 3 - 4 | 5 | 5.0 |  |
| ≥ 5 | 0 | 0.0 |  |
