# Supplementary material for: Anabolic Androgenic Steroid Use Patterns and Steroid Use Disorders in a Sample of Male Gym Visitors
Source: Eur Addict Res. 2023 Feb 2;29(2):99–108. doi: 10.1159/000528256 (PMC10273855; doi:10.1159/000528256)
Supplement: Supplementary file 4 — Supplementary data [file ear-0029-0099-s04.docx]

**Table S4.** Mental health in male anabolic-androgenic steroid (AAS) consumers (N=103).

| Variable | N | % | Mean (SD) |
| --- | --- | --- | --- |
| Satisfaction with physical appearance |  |  |  |
| Very dissatisfied | 5 | 5.0 |  |
| A little dissatisfied | 19 | 19.0 |  |
| Fairly satisfied | 61 | 61.0 |  |
| Very satisfied | 15 | 15.0 |  |
| Mental disorder (any) (lifetime; n=98) ^#^ | 40 | 40.8 |  |
| Mental disorder (lifetime; n=98) ^§^ |  |  |  |
| Depression | 18 | 18.4 |  |
| Anxiety | 18 | 18.4 |  |
| Drug dependence | 15 | 15.3 |  |
| ADHD | 12 | 12.2 |  |
| Number of mental disorders (n=40) ^¥^ |  |  | 0.0 (0.0 – 1.0) |
| Mental disorder arose before the use of AAS | 30 | 75.0 |  |
| Mental disorder arose during the use of AAS | 2 | 5.0 |  |
| Mental disorder arose after the use of AAS | 8 | 20.0 |  |
| Mental well-being score during AAS cycle ^##^ |  |  | 7.69 (1.94) |
| Mental well-being score after AAS cycles ^##^ |  |  | 6.00 (1.84) |
| Consulted a psychiatrist or psychologist (n=97) ^###^ | 12 | 12.4 |  |
| Physically or sexually abused (lifetime; n=98) | 6 | 6.1 |  |
| Sentenced to prison (ever; n=98) | 3 | 3.1 |  |
| ^§^ from a list of seven conditions/disorders, more than one answer possible; ^¥^ median value (interquartile range); ^#^ selected from a list of seven mental disorders; ^##^ for participants that used AAS in a cyclic pattern (n =48), scale from 0 (very bad) to 10 (very good); ^###^ last 12 months. | | | |
