# Supplementary material for: Anabolic Androgenic Steroid Use Patterns and Steroid Use Disorders in a Sample of Male Gym Visitors
Source: Eur Addict Res. 2023 Feb 2;29(2):99–108. doi: 10.1159/000528256 (PMC10273855; doi:10.1159/000528256)
Supplement: Supplementary file 5 — Supplementary data [file ear-0029-0099-s05.docx]

**Table S5.** Correlations between AAS use disorder (AASUD) criteria sum score and AASUD diagnosis (DSM-5 ≥ 4 criteria) with potential predictors.

| Variable | Age | AASUD criteria sum score | AASUD diagnosis  (≥ 4 criteria) | AAS dose (mg/wk.) over last 12 mo. | AAS duration of use  (wks./last 12 mo.) | AAS dose (mg/wk.) during AAS use in last 12 mo. | AAS side-effects (no. over last 12 mo.) | Mental health disorder (any, lifetime) | Physical or sexual abuse (lifetime) | Psychoactive substance dependence (lifetime) | Psychoactive substance use (no. over last 12 mo.) | IPEDs other than AAS (no. over last 12 mo.) |
| --- | --- | --- | --- | --- | --- | --- | --- | --- | --- | --- | --- | --- |
| Age | 1 | -.135 | -.028 | .038 | -.031 | .024 | -.260** | .018 | .178 | .227* | -.176 | -.112 |
| AASUD criteria sum score |  | 1 | .834** | .393** | .246* | .314** | .393** | .243* | -.110 | .193 | .133 | .005 |
| AASUD diagnosis  (≥ 4 criteria) |  |  | 1 | .278** | .249* | .172 | .217* | .236* | -.141 | .187 | -.036 | -.119 |
| AAS dose (mg/wk.) over last 12 mo. |  |  |  | 1 | .453** | .877** | .241* | .164 | -.035 | .161 | .086 | -.196* |
| AAS duration of use (wks./last 12 mo.) |  |  |  |  | 1 | .086 | .285** | .188 | .037 | .060 | -.039 | .188 |
| AAS dose (mg/wk.) during AAS use in last 12 mo. |  |  |  |  |  | 1 | .145 | .126 | -.084 | .125 | .101 | .042 |
| AAS side-effects (no. over last 12 mo.) |  |  |  |  |  |  | 1 | .352** | .045 | .085 | .275** | .040 |
| Mental health disorder (any, lifetime) |  |  |  |  |  |  |  | 1 | .038 | .256* | .049 | .028 |
| Physical or sexual abuse (lifetime) |  |  |  |  |  |  |  |  | 1 | .190 | .128 | -.119 |
| Psychoactive substance dependence (lifetime) |  |  |  |  |  |  |  |  |  | 1 | .265* | .031 |
| Psychoactive substance use (no. over last 12 mo.) |  |  |  |  |  |  |  |  |  |  | 1 | .124 |
| IPEDs other than AAS (no. over last 12 mo.) |  |  |  |  |  |  |  |  |  |  |  | 1 |
| Pearson correlation coefficients are given; AAS = anabolic-androgenic steroids; * correlation significant at p < .05; ** correlation significant at p < 01. | | | | | | | | | | | | |
