# Supplementary material for: Anabolic Androgenic Steroid Use Patterns and Steroid Use Disorders in a Sample of Male Gym Visitors
Source: Eur Addict Res. 2023 Feb 2;29(2):99–108. doi: 10.1159/000528256 (PMC10273855; doi:10.1159/000528256)
Supplement: Supplementary file 6 — Supplementary data [file ear-0029-0099-s06.docx]

**Table S6.** Logistic regression analyses predicting AAS use disorder (AASUD; DSM-5 criteria ≥ 4).

1. Average AAS dose (mg/wk.) during AAS use in the last 12 months as predicting variable.

| Variable | ß | SE ß | Wald’s χ^2^ | df | Eß (OR) | 95% CI |  |
| --- | --- | --- | --- | --- | --- | --- | --- |
| Age (yrs.) | -0.005 | 0.028 | 0.030 | 1 | 0.995 | 0.942-1.052 |  |
| AAS dose (mg/wk.) during AAS use ^a^ over 12 months | 0.001 | 0.000 | 2.414 | 1 | 1.001 | 1.000-1.001 |  |
| Lifetime mental disorder | 0.362 | 0.224 | 2.608 | 1 | 1.437 | 0.962-2.230 |  |
| Constant | -1.769 | 0.968 | 3.337 | 1 | 0.171 |  |  |
| ^a^ for AAS consumers with a ‘blast & cruise’ use pattern, this is the average of the dose during a ‘cruise’ and during a ‘blast’, taking into account their respective duration; * significant at p <. 0.05; Nagelkerke R^2^ for this model: 0.138.   1. Duration of AAS use (weeks) and average AAS dose (mg/wk.) during AAS use in the last 12 months as predicting variables.  \| Variable \| ß \| SE ß \| Wald’s χ^2^ \| df \| Eß (OR) \| 95% CI \| \| --- \| --- \| --- \| --- \| --- \| --- \| --- \| \| Age (yrs.) \| -0.004 \| 0.030 \| 0.020 \| 1 \| 0.996 \| 0.938-1.057 \| \| AAS duration (wks.) over 12 mo. \| 0.031 \| 0.015 \| 4.126 \| 1 \| 1.032* \| 1.001-1.063 \| \| AAS dose (mg/wk.) during AAS use ^a^ over 12 months \| 0.001 \| 0.000 \| 2.073 \| 1 \| 1.001 \| 1.000-1.001 \| \| Lifetime mental disorder \| 0.284 \| 0.229 \| 1.539 \| 1 \| 1.328 \| 0.848-2.080 \| \| Constant \| -2.804 \| 1.166 \| 5.781 \| 1 \| 0.061 \|  \| \| ^a^ for AAS consumers with a ‘blast & cruise’ use pattern, this is the average of the dose during a ‘cruise’ and during a ‘blast’, taking into account their respective duration; * significant at p <. 0.05; Nagelkerke R^2^ for this model: 0.149.  AAS = anabolic-androgenic steroids; DSM-5 = Diagnostic and Statistical Manual of Mental Disorders (5th ed.); OR = odds ratio; SE = standard error; N = 98 (No AASUD = 74, AASUD = 24) due to missing cases (n=5) for “lifetime mental disorder”. \| \| \| \| \| \| \| | | | | | | | |
